# Small extracellular vesicle non-coding RNAs in pancreatic cancer: molecular mechanisms and clinical implications

**DOI:** 10.1186/s13045-021-01149-4

**Published:** 2021-09-08

**Authors:** Moritz Reese, Sameer A. Dhayat

**Affiliations:** Department of General, Visceral and Transplant Surgery, University Hospital Muenster, Albert-Schweitzer-Campus 1 (W1), 48149 Muenster, Germany

**Keywords:** Pancreatic cancer, Pancreatic ductal adenocarcinoma, Exosome, Small extracellular vesicle, Non-coding RNA, MicroRNA, Long non-coding RNA, Circular RNA

## Abstract

Pancreatic cancer has the worst prognosis among common tumors which is attributed to its aggressive phenotype, diagnosis at advanced, inoperable stages, and resistance to systemic therapy. Non-coding RNAs (ncRNAs) such as microRNAs, long non-coding RNAs, and circular RNAs have been established as important regulators of gene expression and their deregulation has been implicated in multiple diseases and foremost cancer. In the tumor microenvironment, non-coding RNAs can be distributed among cancer cells, stromal cells, and immune cells via small extracellular vesicles (sEVs), thereby facilitating intercellular communication and influencing major cancer hallmarks such as angiogenesis, evasion of the immune system, and metastatic dissemination. Furthermore, sEV-ncRNAs have shown promising potential as liquid biopsies with diagnostic and prognostic significance. In this review, we summarize the role of sEVs as carriers of ncRNAs and underlying molecular mechanisms in pancreatic cancer. Moreover, we review the potential of sEV-ncRNAs as biomarkers and highlight the suitability of sEVs as delivery vehicles for ncRNA-based cancer therapy.

## Background

Pancreatic cancer (PC) remains to be the deadliest malignancy among common tumors with pancreatic ductal adenocarcinoma (PDAC) accounting for the vast majority of pancreatic neoplasms. It represents the third-leading cause of cancer-related deaths in the US and with growing incidence poses a major threat to public health. The majority of patients with PDAC present with metastatic disease, for which five-year survival is as low as 3% [1]. Moreover, patients rapidly develop resistance to conventional chemotherapeutic regimens, while surgery poses the only potentially curative treatment. Nonetheless, many patients suffer tumor recurrence following successful resection of PDAC, while surgery itself is complicated and often associated with postoperative complications such as the development of pancreatic fistulae, delayed gastric emptying, chyle leak, and hemorrhage, all of which have a significant impact on patients’ outcome and quality of life [2]. With five-year relative survival rates for localized tumors being as high as 39%, establishing biomarkers for reliable diagnosis—especially at early stages—has become a promising strategy in the fight against PDAC [1]. As such, deregulated non-coding ribonucleic acids (ncRNAs) have proven to be valuable as liquid biopsies in multiple tumor entities, while simultaneously holding the potential to influence major cancer hallmarks at the molecular level [3–5].

As opposed to messenger RNAs (mRNAs), ncRNAs are transcripts of DNA that do not translate into proteins themselves but rather ensure the smooth functioning and regulation of protein biosynthesis. Today, many ncRNAs have been identified that have been attributed multiple functions vital for cellular homeostasis. Transfer RNAs (tRNAs) and ribosomal RNAs (rRNAs) are long-known ncRNAs essential for the translation of mRNA into proteins. In contrast, small nuclear RNAs (snRNAs) associate with proteins to form small nuclear ribonucleoproteins (snRNPs), five of which combine with several accessory proteins to form the spliceosome. The spliceosome in turn processes pre-mRNA by removing non-coding introns to produce mature mRNA that solely consists of protein-coding exons [6]. Given the importance of alternative splicing in cancer, a mechanism frequently hijacked by tumor cells to generate specific transcripts of mature mRNA that benefit tumor progression, an involvement of snRNAs in tumor progression seems apparent [7]. Indeed, snRNA mutations have been reported in multiple types of cancer including medulloblastoma, chronic lymphocytic leukemia, B cell non-Hodgkin lymphomas, as well as hepatocellular and pancreatic carcinoma [8].

Small nucleolar RNAs (snoRNAs) have a length of 60 to 300 nucleotides. They primarily localize in the nucleolus and have been implicated in post-transcriptional modification of ncRNAs such as tRNAs, rRNAs, and snRNAs, vital for the assembly of ribosomes. However, their deregulation has also been observed in diseases such as cancer [9, 10]. For example, tumor-suppressive snoRNAs SNORD50A and SNORD50B can inhibit K-Ras but are frequently deleted in multiple human cancers including melanoma, ovarian, liver, lung, breast, and prostate cancer [11]. In contrast, SNORA23 is commonly overexpressed in PDAC and inversely correlated with prognosis of patients [12].

In this review, we will mostly focus on microRNAs (miRNAs), long non-coding RNAs (lncRNAs), and circular RNAs (circRNAs). Among these, miRNAs have been studied most extensively in the context of medical research. miRNAs are short ncRNAs, approximately 22 nucleotides in length, that as part of the cytosolic RNA-induced silencing complex (RISC) act as key negative regulators of protein expression at the post-transcriptional level [13]. Due to their semi-specific nature, one miRNA can target up to hundreds of mRNAs, while one mRNA can also be the target of many miRNAs [14]. Either way, miRNA-dependent inhibition of gene expression is believed to be mediated mainly through two mechanisms, depending on the grade of specificity between the interacting miRNAs and mRNAs: (1) mRNA cleavage and (2) translational repression [14].

lncRNAs are defined as ncRNAs that exceed a length of 200 nucleotides. Researchers have uncovered that lncRNAs can influence gene expression at multiple levels and their modes of action are much more diverse than that of miRNAs. These mechanisms include (1) epigenetic changes by chromatin interactions, (2) inhibition or promotion of transcription, as well as (3) regulation of gene expression at the post-transcriptional level. A common example for the latter is the sponging of miRNAs, by which lncRNAs assume the role of competitive endogenous RNAs (ceRNAs) that prevent miRNAs from interacting with their target mRNAs [15, 16]. This enables lncRNAs to indirectly influence mRNA translation. By inhibiting oncogenic and tumor-suppressive miRNAs, lncRNAs can exert both tumor-suppressive and oncogenic functions.

Finally, circRNAs are ncRNAs with a loop-like structure that is a result of their biogenesis’s nature. circRNAs emerge from a process termed “backsplicing,” a splicing event in which two splice sites are covalently linked to each other, providing these molecules with their characteristic ring structure, which implies high stability due to protection from exonuclease activity [17]. As circRNAs are a relatively new field of research, many of their functions have yet to be uncovered. It is believed however that circRNAs—similarly to lncRNAs—may serve as miRNA- and protein sponges, thus holding oncogenic as well as tumor-suppressive potential [18].

## Extracellular vesicles

While ncRNAs are abundantly expressed intracellularly, many ncRNAs can also be found in various types of bodily fluids, especially in the blood. Here, a large percentage of ncRNAs is encapsulated within extracellular vesicles (EVs). EVs are a heterogeneous population of non-proliferating nano- and microvesicles with a lipid bilayer that are actively released from almost all cell types and that have been attributed important roles in intercellular communication. EVs can be further classified into exosomes (~ 50–150 nm diameter) and ectosomes (~ 100–1000 nm diameter) that differ from each other in terms of size, biogenesis, and content [19].

**Fig. 1:**
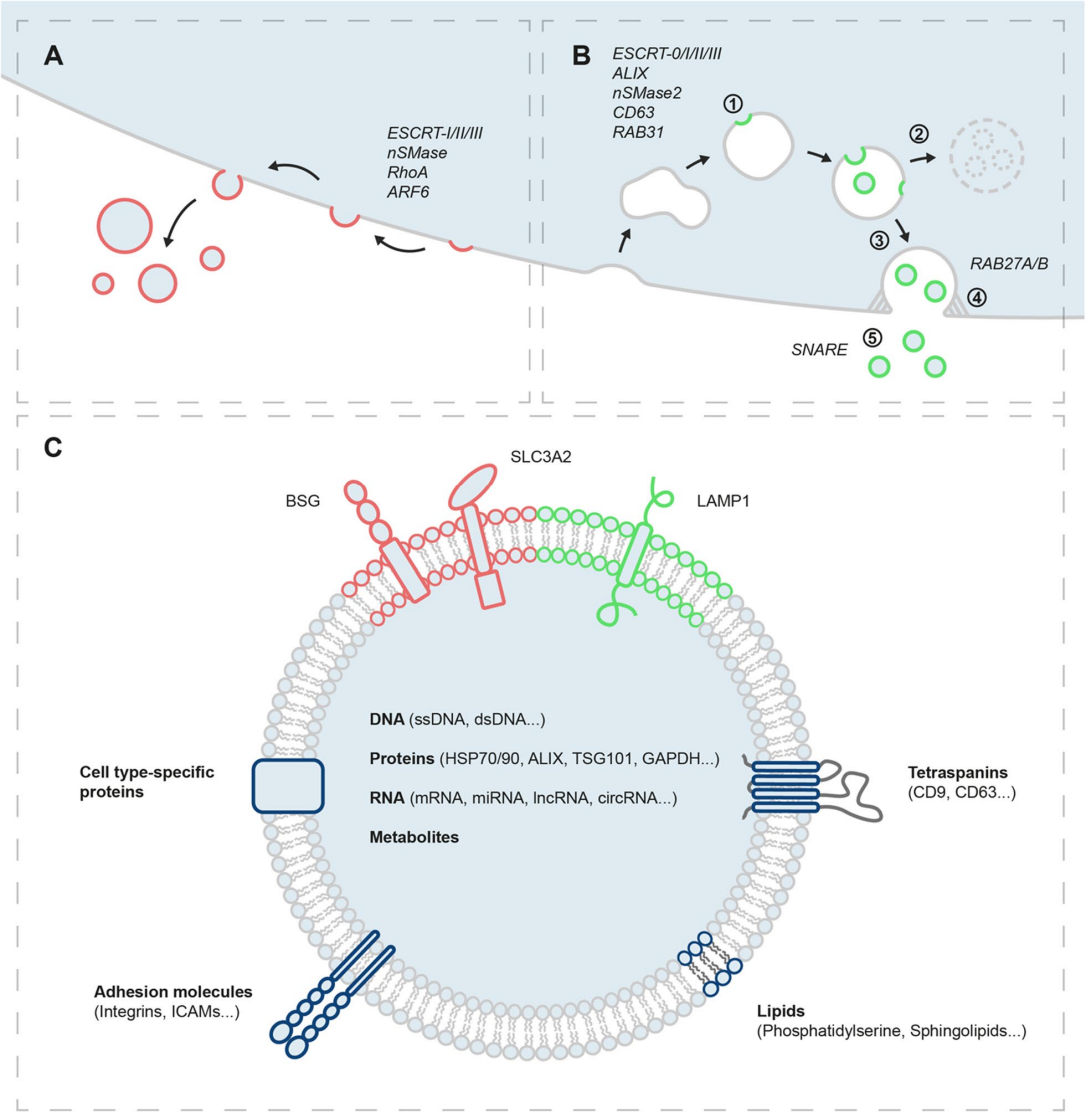
Biogenesis, content, and surface marker profile of small extracellular vesicles. **A** Ectosomes are shed from the plasma membrane by outward budding; **B** (1) Intraluminal vesicles form within endosomes by invagination of the endosomal membrane. Multivesicular bodies (2) fuse with lysosomes to undergo degradation or (3) are directed to the plasma membrane along microtubules. (4) Multivesicular bodies dock at the plasma membrane. (5) Intraluminal vesicles are released into the extracellular space as exosomes upon fusion of multivesicular bodies with the plasma membrane; **C** Content and surface markers of small extracellular vesicles. BSG/SLC3A2 and LAMP1 might represent specific markers for small ectosomes and exosomes, respectively

Exosomes are of endosomal origin: during maturation of endosomes into multivesicular bodies (MVBs), intraluminal vesicles (ILVs) are enriched within MVBs by inward budding. Upon fusion of MVBs with the cell membrane, ILVs are released into the extracellular space as exosomes, loaded with proteins, RNA, DNA, and metabolites (Fig. 1B) [20]. The formation of ILVs within MVBs is orchestrated either in an ESCRT-dependent (endosomal sorting complex required for transport) or ESCRT-independent way. The former is mainly catalyzed by protein complexes ESCRT-0/I/II/III with the help of auxiliary proteins such as ALIX, while the latter can be driven by neutral sphingomyelinase type 2, tetraspanin CD63, or RAB31 [21–24]. In a process incompletely understood, some MVBs undergo degradation by fusing with lysosomes, while others are directed to the plasma membrane (PM), likely utilizing the microtubule system [25]. Finally, RAB27A and RAB27B facilitate docking of MVBs at the PM, while fusion of MVBs with the PM to release ILVs is most likely mediated by SNARE proteins [26–28]. As opposed to exosomes, ectosomes are directly shed from the PM by outward budding (Fig. 1A). Given the similarity to ILV formation within MVBs, it seems plausible that biogenesis of ectosomes also relies on members of the ESCRT protein family but could similarly be driven by sphingomyelinases [29, 30]. Other studies have suggested that ectosome budding from the PM is regulated by small GTPases such as RhoA or ARF6 [31, 32].

Several protocols have been proposed for the isolation of EVs. Conventional methods such as differential (ultra-)centrifugation, size exclusion chromatography, density gradient ultracentrifugation, ultrafiltration, precipitation- and affinity-based capturing methods remain the most common approaches in descending order of popularity [33]. However, microfluidics, tangential flow, as well as field flow fractionation have recently been explored for use in EV isolation and researchers’ interest in these methods has gradually increased over the last few years [33]. While no perfect protocol for EV isolation exists, each technique has its advantages and disadvantages which have been reviewed in detail elsewhere [34, 35]. Importantly, however, none of the aforementioned approaches has been able to reliably separate exosomes from small ectosomes (< 200 nm). In a position paper, the *International Society of Extracellular Vesicles* has therefore advised researchers to use the term ‘small extracellular vesicle’ (sEV) when reporting on studies about exosomes or other small microvesicles, which includes both types of EVs and which we have adopted in this review [36]. Moreover, authors have proposed minimal requirements for the separation, characterization, and conduction of functional studies on EVs in an attempt to further standardize and increase the reproducibility of EV research [36]. Interestingly, Théry and colleagues have recently identified potential surface markers specific for exosomes (LAMP1) and PM-shed ectosomes (BSG, SLC3A2), which could pave the way for separate investigation of these entities in the future (Fig. 1C) [37].

Carrying multiple types of RNA, DNA, proteins, lipids, and metabolites, sEVs have been established as important mediators of intercellular communication [38, 39]. This is enabled by the fact that cargo loading into sEVs—and especially that of RNA—does not occur at random but is an actively regulated process. Indeed, multiple studies have recently shown the importance of RNA-binding proteins in recruiting both mRNA and ncRNAs to sEVs [40]. For example, it was shown that hnRNPA2B1 facilitates enrichment of oncogenic lncRNA-LNMAT2 in bladder cancer-derived sEVs, ultimately promoting lymph node metastasis, while IGF2BP1 was suggested to affect the protein, mRNA, and miRNA composition of melanoma-derived EVs in a way that promotes metastatic dissemination [41, 42]. Moreover, major vault protein (MVP) assists in discharging tumor-suppressive miRNA-193a from colon cancer cells via sEVs, hence promoting tumor progression, while AUF1-mediated recruitment of lncRNA-AFAP1-AS1 to breast cancer-derived sEVs is associated with trastuzumab resistance [43, 44]. Despite major challenges in standardizing research on EVs, this tissue-specific composition and heterogeneity make sEVs attractive targets as biomarkers and therapeutics in many areas of biomedical research including cancer research [45, 46]. In this review, we will focus on (1) the involvement of sEV-derived ncRNAs (sEV-ncRNAs) in tumor biology and progression of PDAC (Table 1), (2) the utility of deregulated sEV-ncRNAs as clinical biomarkers, as well as (3) the potential of sEVs as delivery vehicles for ncRNA-based therapy in PDAC.

**Table 1:** Landscape of non-coding RNAs transmitted by small extracellular vesicles in pancreatic cancer, induced molecular pathways and effect on recipient cells

ncRNA	Transmission by small extracellular vesicles		Target	Downstream signaling/molecular mechanism	Effect in recipient cells	Ref
	from…	to…				
*microRNAs*						
21-5p	TAM	PDAC	KLF3		Stemness, invasion, migration	[83]
	PSCs	PDAC		Induction of Ras/ERK/Akt	Proliferation, migration	[106]
27a	PDAC	Endothelial cells	BTG2		Angiogenesis, tumor growth	[114]
106b	PSCs	PDAC	TP53INP1		GEM resistance	[99]
125b-5p	Highly malignant PDAC	Less malignant PDAC	STARD13	Induction of MEK2/ERK2	Invasion, migration, EMT	[48]
143	hMSCs	PDAC	lncRNA-RP11-363N22.3		Promotes apoptosis, inhibits proliferation, invasion, migration	[102]
145	Primary tumor stroma	PDAC			Apoptosis	[101]
146a	GEM-treated CAFs	PDAC		Induction of Snail	GEM resistance	[100]
155	Pancreatic fibroblasts	CAFs	TP53INP1		Conversion of PSCs into CAFs	[98]
	TAM	Endothelial cells	E2F2		Angiogenesis, tumor growth	[113]
	GEM-resistant PDAC	PDAC	TP53INP1		GEM resistance	[127]
	GEM-treated PDAC	PDAC	DCK		GEM resistance	[126]
203	PDAC	Dendritic cells	TLR4	Inhibition of TNF-α/IL-12 expression	Immunosuppression	[72]
210	GEM-treated CSCs	regular PDAC		Induction of mTOR	Stemness, GEM resistance	[131]
212-3p	PDAC	Dendritic cells	RFXAP	Inhibition of MHC II expression	Immunosuppression	[72]
221-5p	TAM	Endothelial cells	E2F2		Angiogenesis, tumor growth	[113]
222	Highly malignant PDAC	Less malignant PDAC	PPP2R2A	Induction of AKT, inhibition of p27 import to the nucleus	Proliferation, invasion, migration	[49]
301a-3p	Hypoxic PDAC	Macrophages	PTEN	Induction of PI3K/AKT/mTOR	M1-to-M2 polarization of macrophages, immunosuppression	[84]
365	TAM	PDAC	BTG2	Induction of FAK/AKT	Proliferation, invasion, migration	[78]
				Increase of intracellular NTPs and CDA	GEM resistance	[77]
494	PDAC	LN stroma cells, lung fibroblasts	CDH17		Promotes pre-metastatic niche	[58]
		PBMCs		Increase of intracellular calcium fluxes	Expansion of MDSCs and immunosuppression	[74]
501-3p	TAM	PDAC		Induction of TGFBR3	Anti-apoptosis, proliferation, stemness, invasion, migration	[82]
		Endothelial cells			Angiogenesis	
542-3p	PDAC	LN stroma cells, lung fibroblasts	CDH17		Promotes pre-metastatic niche	[58]
616-3p	hypoxic PSCs	PDAC	PTEN	Induction of AKT	Proliferation, invasion, migration	[107]
1260a	PDAC	PBMC		Increase of intracellular calcium fluxes	Expansion of MDSCs and immunosuppression	[74]
1290	PDAC	PSCs		Induction of ERK/Akt	Conversion of PSCs into profibrotic CAFs	[97]
3607-3p	natural killer cells	PDAC	IL-26		Inhibits proliferation, invasion, migration	[76]
4465	hypoxic PSCs	PDAC	PTEN	Induction of AKT	Proliferation, invasion, migration	[107]
5703	PSCs	PDAC	CMTM4	Induction of PAK4/PI3K/Akt	Proliferation	[108]
*Long non-coding RNAs*						
CCAT1	PDAC	Endothelial cells	miR-138-5p	Induction of HMGA1	Angiogenesis, tumor growth	[115]
HULC	PDAC	PDAC			EMT, invasion, migration	[56]
SBF2-AS1	TAM	PDAC	miR-122-5p	Induction of XIAP	Proliferation, invasion, migration	[79]
Sox2ot	Highly malignant PDAC	Less malignant PDAC	miR-200 family	Induction of Sox2	Stemness, EMT, metastasis	[53]
UCA1	Hypoxic PDAC	Endothelial cells	miR-96-5p	Induction of AMOTL2/ERK1/2	Angiogenesis, tumor growth	[116]
*Circular RNAs*						
0000069	PDAC	Benign pancreas		Induction of STIL	Cell cycle progression, proliferation, migration	[59]
0030167	BM-MSCs	PDAC	miR-338-5p	Induction of Wif1, inhibition of Wnt8/β-catenin	Inhibits proliferation, invasion, migration, stemness	[103]
IARS	PDAC	Endothelial cells	miR-122		Endothelial permeability, metastasis	[57]
PDE8A	Highly malignant PDAC	Less malignant PDAC	miR-338	Induction of MACC1/MET/ERK/AKT	Proliferation, invasion, metastasis	[47]
ZNF91	Hypoxic PDAC	Normoxic PDAC	miR-23b-3p	Induction of SIRT1 and glycolysis	GEM resistance	[133]

BM-MSC, bone marrow mesenchymal stem cell; CAF, cancer-associated fibroblast; CDA, cytidine deaminase; CSC, cancer stem cell; DCK, deoxycytidine kinase; EMT, epithelial-to-mesenchymal transition; GEM, gemcitabine; hMSC, human mesenchymal stem cell; LN, lymph node; NTP, nucleotide triphosphate; PBMC, peripheral blood mononuclear cell; PSC, pancreatic stellate cell; Ref., reference; TAM, M2-polarized tumor-associated macrophage

## Influence of sEV-ncRNAs on PDAC at the molecular level

### Proliferation, invasion, EMT, and metastasis

**Fig. 2:**
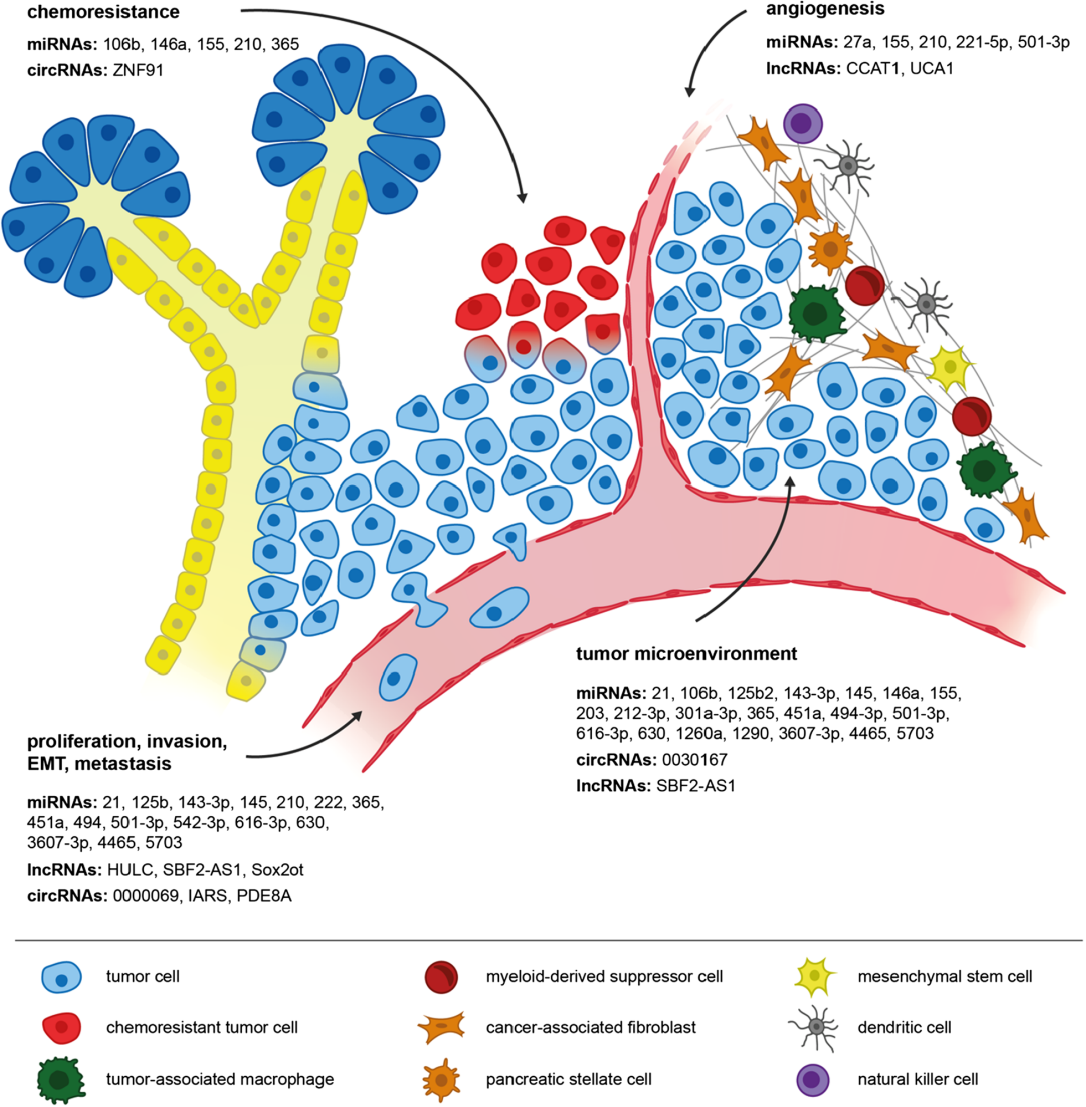
Overview of sEV-ncRNAs involved in the regulation of major characteristics of PDAC, such as chemoresistance, angiogenesis, invasion, metastatic dissemination, as well as communication with immune and stromal cells of the tumor microenvironment

As mediators of intercellular communication, sEVs are utilized by tumor cells to distribute oncogenic ncRNAs in the tumor microenvironment (TME) and several studies have been dedicated to unraveling the molecular mechanisms by which sEV-ncRNAs affect the proliferation, invasion, and migration of PDAC (Fig. 2). For example, circRNA-PDE8A was introduced as an oncogenic ncRNA in PDAC that can be transmitted between PDAC cells via sEVs [47]. In recipient cells, circRNA-PDE8A acts as a sponge to miR-338, which induces expression of MACC1, a positive transcriptional regulator of receptor tyrosine kinase MET. Overexpression of MET promotes tumor progression via activation of downstream AKT/ERK signaling [47].

Similarly, oncogenic characteristics of miRNAs 222 and 125b-5p were attributed to interference with AKT or ERK signaling, too. Following sEV-encapsulated delivery to PDAC cells, miRNA-222 activates the PPP2R2A/AKT pathway, resulting in an accumulation of p27 in the cytoplasm that promotes PDAC cell proliferation, invasion, and metastatic dissemination, while miRNA-125b-5p inhibits tumor suppressor STARD13, thus activating the MEK/ERK pathway [48, 49]. Controversially, miRNA-125b-5p transmission via sEVs has also been shown to promote tumor immunity in PDAC (discussed below), potentially compromising its clinical utility as a therapeutic target [50].

Epithelial-to-mesenchymal transition (EMT) is a process, by which epithelial cancer cells gain mesenchymal cell-like characteristics. It is characterized by a loss of intercellular junctions, cytoskeletal remodeling and hence loss of apical-basal cell polarity, as well as detachment from and invasion beyond the basement membrane [51]. Activation of EMT initiates a transcriptional program that not only facilitates metastasis but is also connected with the induction of a cancer stem cell-like state, implicating resistance to chemotherapy and promoting tumor recurrence [52]. Li and colleagues found that lncRNA-Sox2ot is overexpressed in PDAC cells exhibiting a highly malignant phenotype. Transmission to adjacent, less malignant PDAC cells via sEVs induces a more aggressive phenotype, as lncRNA-Sox2ot acts as a sponge to members of the miRNA-200 family, a potent suppressor of EMT [53, 54]. Inhibition of miRNA-200 induces transcription factor Sox2, which (1) promotes EMT and cancer stemness in vitro as assessed by characteristic protein markers and (2) increases the number of liver metastases in vivo [53].

Similar results were observed for lncRNA-HULC. Takahashi and colleagues found that TGF-β causes overexpression of lncRNA-HULC within PDAC sEVs [55, 56]. Meanwhile, horizontal transfer of lncRNA-HULC induces EMT in recipient PDAC cells [55, 56]. It should be noted, however, that PANC-1 cells served as both sEV donors and recipients. Moreover, incubation of PANC-1 cells with control sEVs had a significant impact on EMT markers, too, suggesting an overdose of sEVs. While this does not contradict an oncogenic role of lncRNA-HULC per se, it remains uncertain if physiological numbers of sEVs carry sufficient amounts of lncRNA-HULC to induce the effects reported above.

Interestingly, PDAC tumor cells can also prime non-malignant tissue to facilitate metastatic dissemination. Researchers showed that circRNA-IARS expression correlates with malignancy of PDAC and that circRNA-IARS is abundant in PDAC sEVs [57]. Following transmission to human umbilical vein endothelial cells, circRNA-IARS inhibits miRNA-122, altering the levels of RhoA, F-actin, and ZO-1 in vitro, suggesting endothelial hyperpermeability and promotion of metastatic spread. Indeed, PDAC xenografts overexpressing circRNA-IARS present with a higher number of liver nodules than those with a regular expression of circRNA-IARS [57]. Moreover, Zöller and colleagues have suggested that PDAC capitalize on sEV miRNAs to support the preparation of a pre-metastatic niche in distant organs. PDAC sEVs were shown to be preferentially internalized by lymph node stromal cells and lung fibroblasts. Subsequently, sEV cargo altered the expression of proteases, adhesion molecules, chemokine ligands, and additional genes related to angiogenesis and cell cycle progression [58]. Mechanistically, this proposed involvement of miRNAs was illustrated for miRNAs 494 and 542-3p which downregulate cadherin-17 in recipient stromal cells [58]. On a final note, researchers recently suggested that sEV-ncRNAs might contribute to the malignant transformation of benign pancreatic tissue. PDAC sEVs were shown to contain high levels of circRNA-0000069 that upon transfer to benign human pancreatic duct epithelial cells induced the expression of transcription factor STIL, entailing enhanced cell proliferation, migration, and cell cycle progression [59]. However, the relevance of this observation remains uncertain due to the experimental setting in which rapidly proliferating tumor cells served as sEV donors. Even so, these findings underline a general oncogenic potential of circRNA-0000069, in line with results acquired in colorectal and cervical cancer [60, 61].

### Tumor microenvironment

**Fig. 3:**
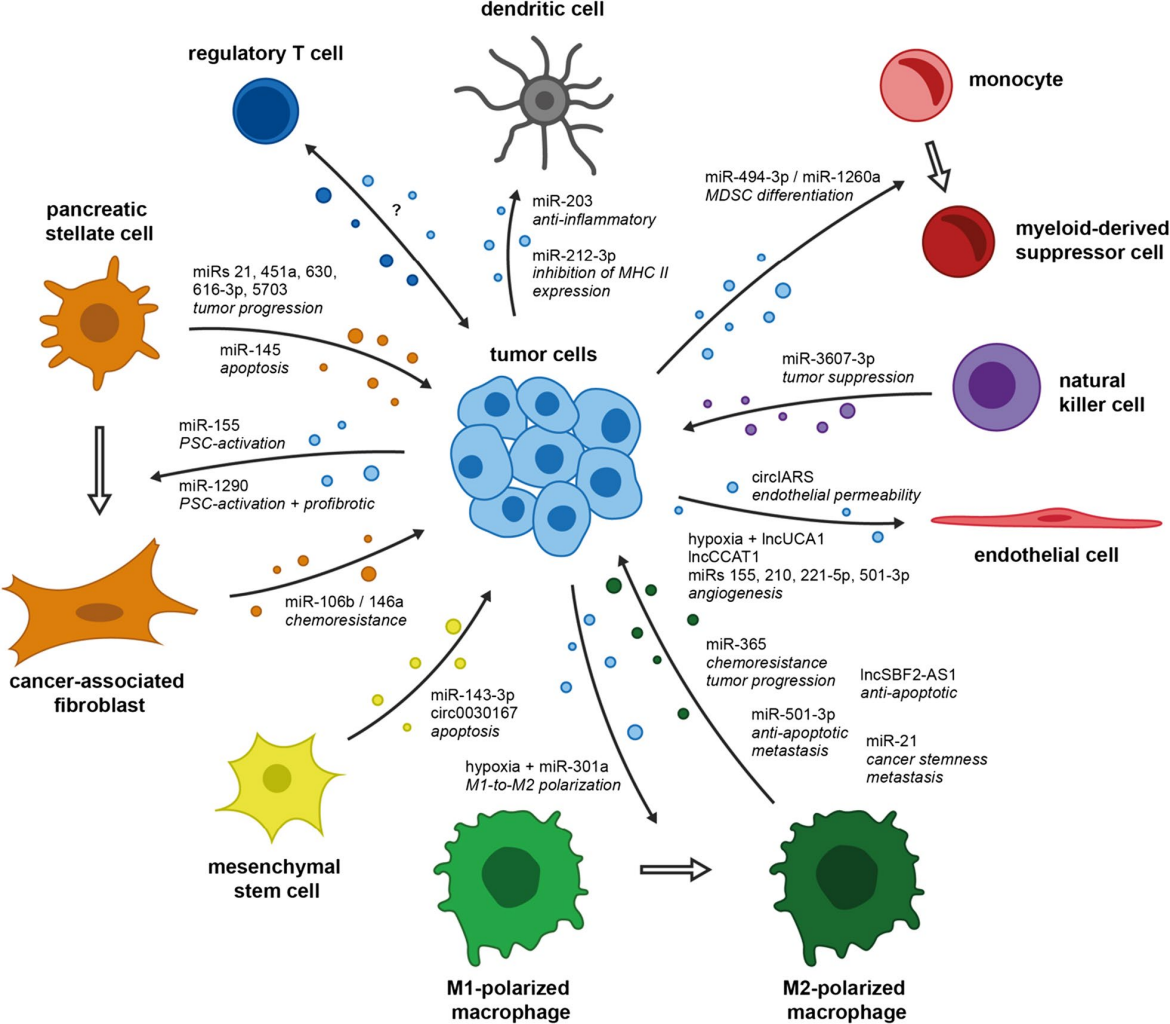
Intercellular communication in the PDAC tumor microenvironment via sEV-ncRNAs

PDAC is characterized by a dense, desmoplastic stroma and immunosuppressive microenvironment that hinders efficient delivery of therapeutics to the tumor and promotes cancer progression. PDAC stroma mainly consists of extracellular matrix proteins that are produced by activated pancreatic stellate cells (PSCs) [62]. In their activated form, PSCs gain fibroblast-like characteristics and have been shown to promote carcinogenesis and tumor progression [63]. Additionally, three main types of immune cells have been linked to the immunosuppressive TME of PDAC: myeloid-derived suppressor cells (MDSCs), regulatory T cells (Tregs), and M2-polarized tumor-associated macrophages (TAMs) exhibiting tumor-promoting activity (Fig. 3) [64–66].

#### Interaction with immune cells

Although immune checkpoint inhibitors have revolutionized the treatment of immunologically ‘hot’ tumors such as melanoma, they have yielded disappointing results in PDAC, regarded to be an immunologically ‘cold’ tumor [67, 68]. While limited response rates are in part attributed to relatively low numbers of somatic mutations, the immunosuppressive TME further contributes to the failure of immunotherapy in PDAC [69].

Accounting for only a small percentage of immune cells of the TME, dendritic cells play a central role in initiating the CD8^+^ and CD4^+^ T cell-mediated immune response that is vital for tumor immunity [70]. However, PDAC cells have been shown to manipulate the pro-inflammatory characteristics of dendritic cells via transfer of sEV-miRNAs. Horizontal transfer of miRNA-203 to dendritic cells inhibits the expression of TLR4, thereby obstructing the production of TNF-α, IFN-β, and IL-12 [71]. Similarly, sEV transfer of miRNA-212-3p from PDAC to dendritic cells inhibits the expression of transcription factor RFXAP that promotes MHC class II gene expression [72]. While these studies provide a mechanism for indirect inhibition of T cell response, it was recently reported that PDAC cells can directly induce apoptosis of T lymphocytes in the TME as well [73]. Intriguingly, the effects were ascribed to tumor cell-derived sEVs, although the involvement of ncRNAs was not specifically reported.

On a different note, Basso et al. showed that PDAC cells can expand the population of immunosuppressive MDSCs among total human peripheral blood mononuclear cells through transfer of sEV-miRNAs 494-3p and 1260a [74]. Interestingly, the effect was further enhanced when using sEVs derived from SMAD4-negative PDAC cells, a tumor suppressor frequently mutated in PDAC and associated with shorter overall survival of patients [74, 75]. In contrast, natural killer cells present an anti-tumorigenic population of immune cells within the TME that secrete sEVs enriched with miRNA-3607-3p and can be transferred to PDAC, thus inhibiting tumor progression through downregulation of IL-26 [76].

Several studies have been dedicated to uncovering the mechanisms by which TAMs affect tumor progression through paracrine signaling via sEVs. For example, miRNA-365 is overexpressed in TAM-derived sEVs. Incubation of sEVs with PDAC cells contributes to (1) gemcitabine (GEM)-resistance (described below) and (2) promotes PDAC proliferation, invasion, and migration by targeting BTG2, thus inducing FAK/AKT signaling [77, 78]. Moreover, Yin et al. discovered that TAMs can transfer lncRNA-SBF2-AS1 to PDAC cells via sEVs [79]. In PDAC cells, lncRNA-SBF2-AS1 acts as a ceRNA to miRNA-122-5p, leading to the consecutive upregulation of XIAP. In line with previous studies, anti-apoptotic characteristics of XIAP facilitate PDAC progression in vitro and in vivo [79, 80].

Aberrant TGF-β signaling is evident in nearly half of all PDAC [81]. Researchers recently discovered that miRNA-501-3p is overexpressed in TAM-derived sEVs [82]. sEVs enhance the proliferative, migratory, and anti-apoptotic nature of PDAC cells in vitro, while injection of TAM sEVs into caudal veins of nude mice harboring PDAC xenografts accelerate tumor growth and increase the number of hepatic and pulmonary metastases [82]. Mechanistically, effects were attributed to miRNA-501-3p inhibiting TGFBR3, thereby inducing the TGF-β signaling pathway [82]. In addition to the aforementioned ncRNAs, TAM sEVs were also shown to overexpress well-known oncogenic miRNA-21 [83]. Horizontal transfer to PDAC cells downregulates KLF3, which promoted the stemness of PDAC cells as assessed by expression levels of Nanog and Oct4, tumorsphere, and colony formation assay [83].

Finally, Wang et al. discovered that hypoxia triggers PDAC cells to secrete sEVs enriched with miRNA-301a-3p, which in turn promotes the polarization of monocytes into M2-polarized TAMs by inhibition of PTEN and activation of PI3K/AKT/mTOR signaling [84]. Subsequently, M2-polarized TAMs improve the metastatic potential of PDAC cells in vivo and in vitro [84]. Of note, Su et al. found that M2-polarized macrophages can also be reprogrammed into a tumor-suppressive M1 phenotype by transfer of sEV-miRNAs 155 and 125b [50]. Although miRNA-125b-encapsulating nanoparticles had later been shown to enhance the impact of paclitaxel in epithelial ovarian cancer through the aforementioned mechanism, clinical trials evaluating miRNA-125b and miR-155 mimics should be conducted with caution, given the oncogenic potential of both molecules [85–87].

Tregs represent the third major fraction of pro-tumorigenic immune cells in the PDAC microenvironment. Recently, Cao and colleagues provided the first evidence that expansion of Tregs in PDAC at least partially relies on sEVs [88]. Even so, an involvement of sEV-ncRNAs remains uncertain, although such observations have been made in several other types of tumors. For example, sarcoma and lung tumor cells were shown to induce Treg population through transmission of miRNA-214, while breast cancer cells promote Treg expansion through horizontal transfer of lncRNA-SNHG16 [89, 90]. Therefore, future studies in PDAC could investigate the extent to which crosstalk between tumor cells and Tregs relies on sEV-ncRNAs. Additionally, no study has yet evaluated the relationship between sEV-ncRNAs and immune checkpoints such as PD-1/PD-L1, although investigations in other types of cancer have yielded promising results. For example, sEV miR-23a-3p promotes PD-L1 expression in macrophages, thereby inhibiting T cell response in hepatocellular carcinoma, while cervical cancer cells escape T cell immunity by sEV miRNA-1468-5p-induced overexpression of PD-L1 on lymphatic vessels [91, 92]. Moreover, expression levels of sEV-miRNAs could be correlated with response to immunotherapy in melanoma [93]. In future, these results could inform the initiation of similar studies in PDAC. Positive results could help (1) selecting PDAC patients suitable for immunotherapy and (2) improve the overall benefit of immunotherapy in PDAC.

#### Interaction with stromal cells

Cancer-associated fibroblasts (CAFs) represent a highly abundant subtype of stromal cells in the PDAC TME and they originate from normal fibroblasts—mostly PSCs—through activating signals such as inflammation, physiological stress, TGF-β, and other receptor tyrosine kinase-binding ligands [94]. CAFs can be further classified into different subtypes such as inflammatory CAFs and myofibroblastic CAFs: while myofibroblastic CAFs are believed to be the main producers of the acellular PDAC stroma, inflammatory CAFs have been shown to promote PDAC progression through secretion of cytokines [95, 96]. Masamune and colleagues found that PSCs co-cultured with PDAC cells can be activated to α-SMA-expressing CAF-like cells, the mechanism proposed being sEV transfer of miRNA-1290, which is overexpressed in PDAC sEVs [97]. sEV transfer of miRNA-1290 also induces ERK and Akt signaling, thereby promoting the proliferative behavior of CAF-like cells as well as the production of procollagen type I C-peptide [97]. Similarly, the transfer of tumor cell-derived sEV miRNA-155 has been shown to promote the conversion of normal fibroblasts into CAF-like cells, as reflected in the expression of α-SMA and fibroblast activation protein [98]. Subsequently, CAF-like cells were shown to enhance the migratory ability of PDAC cells [98]. Finally, CAFs also participate in promoting GEM resistance through transfer of sEV-miRNAs 106b and 146a (discussed below) [99, 100].

While initially believed to be solely pro-tumorigenic, the understanding of the PDAC TME now is much more complex, as researchers have uncovered the co-existence of both tumor-promoting as well as tumor-suppressive stromal cell types. This is supported by the finding that primary tumor-derived stromal fibroblasts secrete sEVs overexpressing miRNA-145, which upon transmission to PDAC cells induce apoptosis [101]. Furthermore, mesenchymal stem cells (MSCs) have been implicated in tumor cell apoptosis, too. Researchers found that MSC-derived sEVs are highly enriched with miRNA-143-3p [102]. Transmission to PDAC cells in vitro induces apoptosis, thus suppressing tumor cell viability, migration, and invasion [102]. Moreover, researchers recently provided evidence that MSC sEVs overexpress circRNA-0030167 as well [103]. Incubation with PDAC cells induces tumor cell apoptosis, as circRNA-0030167 inhibits miRNA-338-5p, thereby increasing the expression of tumor suppressor WIF1 [103].

On the pro-tumorigenic side, miRNAs 21, 616-3p, 4465, and 5703 are overexpressed in PSC sEVs and horizontal transfer of these miRNAs can induce a more malignant phenotype in recipient PDAC cells [104–107]. For example, sEV transfer of miRNA-21 induces EMT and consequently facilitates cell migration of PDAC cells in vitro through activation of Ras/ERK and Ras/Akt signaling [104]. Moreover, miRNA-5703 downregulates CMTM4, thereby lifting the inhibition of PAK4 which entails activation of PI3K/Akt signaling and increases PDAC cell proliferation and viability [108]. Overexpression of miRNAs 616-3p and 4465 in PSC sEVs can be further enhanced by culturing PSCs under hypoxic conditions and similarly to miRNA-5703, transfer to PDAC cells results in a more malignant PDAC phenotype through inhibition of tumor suppressor PTEN and activation of the PI3K/AKT pathway [107]. Transmission of miRNAs 451a and 630 between PSCs and PDAC cells has also been speculated to contribute to the malignant phenotype of PDAC, as these two miRNAs were found to be selectively overexpressed in PSC-derived sEVs [106]. Due to the study design, however, the functional changes observed in PDAC cells could only be attributed to PSC-derived sEVs in general but not to sEV-miRNAs 451a and 630, specifically [106].

Great progress has been made in unraveling the molecular mechanisms of the complex microenvironment of PDAC. Owing to an incomplete understanding of their pro- as well as anti-tumorigenic functions, initial attempts at therapeutically targeting the cellular as well as acellular components of the TME have failed [109, 110]. In light of an improved comprehension of the PDAC TME, future clinical trials targeting CAFs and immune cells promise to be more successful in improving the outcome of patients with PDAC. Disrupting their communication via sEVs should be evaluated as part of a multi-modal treatment approach that will be necessary to overcome the lethal disease that is PDAC.

#### Angiogenesis and interaction with endothelial cells

As rapidly proliferating tissues, malignant tumors require sufficient vascularization to ensure a constant supply of nutrients and oxygen for continuous growth and progression. Angiogenesis has therefore been identified as a hallmark of cancer and a promising target in cancer therapy, equally [111, 112]. In PDAC, both tumor cells and TAMs have been shown to promote angiogenesis via transfer of sEV-ncRNAs. For example, Yin et al. shows that TAM-derived sEVs are highly enriched in miRNA-501-3p [82]. Incubation of human microvascular endothelial cells with TAM sEVs promotes angiogenesis as reflected in (1) protein levels of VEGFA, VEGFR2, ANG2, and PlGF, and (2) enhanced angiogenic ability of human microvascular endothelial cells in vitro [82]. TAM sEVs also overexpress miRNAs 155-5p and 221-5p [113]. Horizontal transfer of miRNAs results in enhanced tube formation ability of mouse aortic endothelial cells, mediated through downregulation of transcription factor E2F2. Interestingly, injection of TAM sEVs also promotes angiogenesis in vivo, as reflected in a significant increase in microvascular density of pancreatic tumors in a murine PDAC xenograft [113].

In addition to TAMs, it has recently been reported that PDAC cells can enhance the angiogenic ability of endothelial cells, too. Mechanistically, this has been attributed to sEV-mediated transfer of miRNA-27a, which downregulates BTG2 [114]. In contrast, knockdown of miR-27a in vivo results in inhibition of tumor growth and reduction of microvascular density [114]. Moreover, lncRNA-CCAT1 is overexpressed in PDAC tissue, cell lines, and sEVs under normoxic conditions. Transfer to human umbilical vein endothelial cells induces their proliferative activity by inhibition of miRNA-138-5p and upregulation of HMGA1 [115]. Although the aforementioned studies certainly improve our understanding of neoangiogenesis in PDAC, it is important to remember that tumor angiogenesis will frequently be driven by environmental factors such as hypoxia. Therefore, replicating these conditions in basic research might help enhance the translational relevance of acquired results. For example, Guo et al. recently explored the impact of hypoxic cell culture conditions on the ncRNA composition of sEVs [116]. Interestingly, authors found that hypoxia leads to an accumulation of lncRNA-UCA1 within PDAC sEVs, which promotes the migratory and angiogenic abilities of human umbilical vein endothelial cells in vitro, and enhances tumorigenesis and angiogenesis in vivo [116]. Mechanistically, lncRNA-UCA1 acts as a ceRNA to miRNA-96-5p, therefore inducing AMOTL2. Upregulation of AMOTL2 activates ERK1/2 signaling, which has previously been implicated in tumor progression and angiogenesis in several cancer types [117, 118].

While anti-angiogenic therapy has been approved for many tumor entities, no such therapy has been successful in improving the prognosis of PDAC patients [119–122]. Several reasons have been identified that might have contributed to the failure of anti-angiogenic therapeutics in PDAC. For example, PDAC is known to be a hypovascular tumor, which also impairs drug delivery to tumor cells in general [123]. Moreover, it has been suggested that ineffective anti-VEGF therapy might actually induce a more malignant PDAC phenotype [124, 125]. However, most clinical trials in PDAC have focused on combining anti-angiogenic therapy with common chemotherapeutic regimens such as GEM [121, 122]. Therefore, novel combinatory therapeutic approaches with complementing mechanisms of action could provide a rationale for future clinical trials, to which targeting sEV-ncRNAs could contribute via the above-reviewed mechanisms.

### Therapeutic resistance

#### Chemotherapy

Limited efficacy of systemic treatment poses a major obstacle in the fight against PDAC and paracrine delivery of sEV-ncRNAs has been shown to contribute to the chemoresistant phenotype of PDAC. In that regard, most studies have focused on the molecular mechanisms behind GEM resistance, the chemotherapeutic agent most frequently administered. For example, it was shown that miRNA-155 is highly enriched in sEVs secreted by GEM-resistant PDAC cells, while transmission to GEM-sensitive PDAC cells induces resistance through downregulation of deoxycytidine kinase (DCK) and TP53INP1 [126, 127]. As DCK is required for the phosphorylation of GEM into its active metabolite GEM-triphosphate, a lack of DCK entails inactivation of GEM by deoxycytidine deaminase [128]. In contrast, TP53INP1 is a proapoptotic tumor suppressor induced by p53 but is negatively regulated by miRNA-155 and downregulated in PDAC [129].

Representing only a small subpopulation of all tumor cells, cancer stem cells have been shown to promote tumor progression as well as chemoresistance in multiple tumors including PDAC [52, 130]. In that regard, Yang et al. provided evidence that PDAC cancer stem cells overexpress sEV miRNA-210 under treatment with GEM. Horizontal transfer to GEM-sensitive PDAC cells upregulates drug resistance-related proteins BCRP, MDR1, and YB-1, impairing the effect of GEM treatment in vitro and in a murine PDAC xenograft [131]. In PDAC, chemoresistance is also driven by hypoxia [132]. Interestingly, it was recently shown that GEM resistance acquired under hypoxia can be transferred to normoxic cells, too. Mechanistically, hypoxia causes overexpression of sEV circRNA-ZNF91, which upon transmission to normoxic cells induces GEM resistance through inhibition of miR-23b-3p and stabilization of HIF-1α [133].

In contrast to PDAC tumor cells themselves, stromal- and immune cells of the TME also contribute to GEM resistance. Under treatment with GEM, CAFs enrich miRs 106b and 146a in sEVs, which trigger chemoresistance in recipient PDAC cells through inhibition of TP53INP1 and induction of Snail [99, 100]. GW4869 treatment (a neutral sphingomyelinase inhibitor that inhibits formation of ILVs within MVBs and hence biogenesis of exosomes) of mice co-injected with PDAC cells and CAFs significantly improved the efficiency of GEM treatment [100]. It should be noted, however, that neutral sphingomyelinases not only participate in biogenesis of exosomes but also in post-Golgi trafficking [134]. Moreover, cytotoxic effects of GW4869 might equally be attributed to interaction with phosphatidylserine as illustrated in myeloma [135]. In addition to CAFs, TAMs have also been shown to contribute to GEM resistance, the proposed mechanism being sEV transfer of miRNA-365 [77]. As opposed to miRNA-155, miRNA-365 induces GEM resistance by upregulating deoxycytidine deaminase, the antagonist of DCK responsible for inactivation of GEM [77, 126].

Altogether, the aforementioned studies underline the ability of ncRNAs to promote GEM resistance. While this has previously been shown in multiple studies, the novelty of the studies summarized above derives from the hypothesis that chemoresistance can be distributed among cancer cells via sEVs. However, sEVs were mostly administered in supraphysiological concentrations. Therefore, future studies could evaluate if ncRNA-delivery by sEVs merely imitates ncRNA mimic transfection or if administration of sEVs at physiological concentrations has a significant impact on tumor biology, too. Moreover, as systemic therapy of PDAC evolves, researchers might also explore an involvement of sEV-ncRNAs in resistance to emerging therapeutic concepts such as FOLFIRINOX (folinic acid, fluorouracil, irinotecan, oxaliplatin).

#### Radiotherapy

While chemotherapy is administered in both the (neo-)adjuvant and palliative setting, radio(chemo)therapy is not routinely offered to PDAC patients, as multiple clinical trials could not prove a significant survival benefit of chemoradiation over chemotherapy in multiple settings [136, 137]. Therefore, further fundamental research and clinical trials are needed to help us understand whether subgroups of patients such as those with borderline-resectable tumors might benefit from radiation [138, 139]. Jiang et al. suggested that sEV miRNA-194-5p might contribute to survival and tumor repopulation of PDAC after radiotherapy [140]. Authors found that irradiated PDAC cells release large numbers of sEVs highly enriched with miRNA-194-5p that upon delivery to tumor repopulating cells inhibit cancer progression. This enables the recovery of PDAC cells from irradiation through induction of DNA damage response [140]. In contrast, sEV miRNA-6823-5p has been suggested to promote radiotherapy-induced DNA damage of PDAC cells by inhibiting superoxide dismutase expression, which plays an important role in regulating intracellular levels of reactive oxygen species [141]. Given the opposing involvement in radiosensitivity suggested by the two studies above, further fundamental research is needed to elucidate if targeting sEV-ncRNAs can enhance the clinical benefit of radiotherapy in PDAC.

## sEV-ncRNAs as liquid biopsies in PDAC

In recent years, multiple research groups have explored the clinical utility of sEVs in various diseases, above all, cancer. As minimally-invasive liquid biopsies, sEV-ncRNAs have since entered the limelight of oncologic research, informing the initiation of multiple studies investigating their diagnostic and prognostic potential in many different tumor entities including PDAC (Tables 2, 3).

**Table 2:** Diagnostic significance of sEV-derived ncRNAs in PDAC

Reference	Sample	sEV isolation	Normalization	ncRNA (expression in PC)		Patients enrolled	Diagnostic accuracy (AUC) (PC vs. non-PC if not stated otherwise)
				(↑)	(↓)		
Que et al. [142]	Serum	UC	RNU6B	miR-17-5p miR-21	–	22 PDAC, 6 BPT, 7 AC, 6 CP, 8 HC	**0.887** (miR-17-5p) **0.897** (miR-21)
Madhavan et al. [156]	Serum	DGUC	RNU6, SNORD43 18S & 5S rRNA	miRs 1246, 4644, 3976, 4306	–	112 PDAC, 11 other PC, 22 BPT, 23 CP, 18 HC	*Not assessed*
Machida et al. [158]	Saliva	TEI	RNU6	miR-1246 miR-4644	–	12 PBC (thereof 9 PDAC), 13 HC	**0.814** (miR-1246), **0.763** (miR-4644) **0.833** (miRs 1246 + 4644)
Lai et al. [146]	Plasma	UC	miR-425-5p	miRs 10b, 20a, 21, 30c, 106b, 181a	miRs let7a, 122	29 PDAC, 11 CP, 6 HC	**1.00** (miRs 10b, 21, 30c, 181a, let7a) **0.99** (miR-122), **0.95** (miR-20a), **0.85** (miR-106b)
Chen et al. [229]	Serum	UC	5S rRNA	miR-23b-3p	–	16 PDAC, 20 HC	**1.00** (miR-23-3p)
Xu et al. [157]	Plasma	EQ	cel-miR-54	miR-1246 miR-196a miR-196b (*n.s.*)	–	15 PC (UICC I-IIa) 15 HC	**0.73** (miR-1246) **0.81** (miR-196a) **0.71** (miR-196b)
Goto et al. [145]	Serum	EQ	*not specified*	miRs 21, 191, 451a	–	32 PC, 22 HC	**0.826** (miR-21), **0.788** (miR-191) **0.759** (miR-451a)
Zhou et al. [152]	Plasma	EQ	miR-103a	miR-122-5p, miR-193b-3p	miR-221-3p	31 PC, 37 HC	**0.772** (miR-122-5p), **0.651** (miR-193b-3p) **0.849** (miRs 122-5p + 193b-3p)
Kitagawa et al. [162]	Plasma	*not specified*	*not specified*	SNORA74A, SNORA25, SNORA22, SNORA14B, SNORD22	–	27 PDAC, 13 HC	**0.909** (SNORA74A) **0.903** (SNORA25) **0.883** (SNORA22) **0.875** (SNORA14B) **0.862** (SNORD22)
Nakamura et al. [160]	PJ	UC	miR-16	miR-21 miR-155	–	27 PDAC, 8 CP	**0.83** (miR-21) **0.89** (miR-155) **0.91** (miR-21 + miR-155)
Zou et al. [230]	Serum	EQ	cel-miR-39	miRs 19a, 19b, 192	–	32 PC, 32 HC	*Not assessed*
Shang et al. [204]	Plasma	UC	RNU6	–	miR-1231	32 PC, 20 HC	*Not assessed*
Sun et al. [76]	Plasma	UC	*not specified*	miR-3607-3p	–	40 PC, 20 HC	*Not assessed*
Takahashi et al. [55]	Serum	EQ	RNU6B	lncHULC	–	20 PDAC, 22 IPMN, 21 HC	**0.92**
Reese et al. [155]	Serum	UC	cel-miR-39	miRs 200b, 200c	–	56 PDAC, 11 CP, 22 HC	**0.77** (miR-200b TSE) **0.68** (miR-200c TSE) **0.97** (4-miR-panel + CA.19-9)
Pu et al. [151]	Plasma	TEI	n/a *	miR-10b, miR-21	–	36 PC, 65 HC	**0.6543** (miR-10b) **0.7171** (miR-21) **0.791** (miR-10b + miR-21)
Yoshizawa et al. [159]	Urine	EQ	miR-8069	ratio of miRs 3940-5p/8069	–	43 PDAC, 12 CP, 25 HC	**0.732**
Yang et al. [167]	Plasma	TENPO	*not specified*	miR-409	–	57 PDAC, 49 HC, 12 CP, 3 IPMN, 12 BPT, 3 others	**0.95** (5-marker-panel including miR-409, 2 mRNAs, ccfDNA, and CA.19-9)
Flammang et al. [231]	Serum	UC	cel-miR-39	miR-192-5p	–	44 PDAC, 11 CP, 12 HC	**0.83** (PDAC vs. HC) **0.54** (PDAC vs. CP)
Wu et al. [144]	Serum	EQ	cel-miR-39	miR-21, miR-210	–	30 PC, 10 CP	**0.83** (miR-21), **0.85** (miR-210) **0.90** (miR-21 + miR-210) **0.9** (miR-21 + CA-19-9) **0.9** (miR-210 + CA.19.9)
Guo et al. [116]	Serum	EQ	β-actin	lncUCA1	–	46 PC, 16 HC	**0.7813**
Zhou et al. [150]	Plasma	MC	CD81	miRs 10b, 21, 451a	–	30 PC, 10 CP	**0.875** (miR-10b) **0.939** (miR-21) **0.930** (miR-451a) **1.00** (miRs 10b + 21 + 451a + EphA2)
Cao et al. [107]	Plasma	TEI	cel-miR-39	miR-616 miR-4465	–	50 PC, 30 HC	*Not assessed*
Wang et al. [232]	Serum	EEM	RNU6B	–	miR-1226	17 PDAC, 12 BPD	**0.74** (PDAC vs. BPD)
Shao et al. [154]	Serum	EQ	cel-miR-39	miR-483-3p	–	63 PDAC, 22 HC	**0.69** (sEV miR-483-3p) **0.84** (serum + sEV miR-483-3p)
Han et al. [115]	Plasma	EQ	RNU6	lncCCAT1	–	93 PC, 93 HC	*not assessed*

AC, ampullary carcinoma; AUC, area under the receiver operating characteristic curve; BPT, benign pancreatic tumor; cel, caenorhabditis elegans; ccfDNA, circulating cell-free DNA; CP, chronic pancreatitis; DGUC, density gradient ultracentrifugation; EEM, ExoEasy Maxi Kit (*Qiagen*); EQ, ExoQuick Exosome Isolation Kit (*System Biosciences*); HC, healthy control; MC, microfluidic chip; mRNA, messenger RNA; other PC, non-PDAC pancreatic cancer; *n.s.*, not significant; PanNET, pancreatic neuroendocrine tumor; PBC, pancreatobiliary cancer; PC, pancreatic cancer (all entities); PDAC, pancreatic ductal adenocarcinoma; PJ, pancreatic juice; TEI, Total Exosome Isolation Kit (*ThermoFisher*); TENPO, track etched magnetic nanopore device; UC, ultracentrifugation

^*^Pu et al. utilized a tethered cationic lipoplex nanoparticles biochip for direct and absolute quantification of sEV microRNA, obviating the need for normalization

**Table 3:** Prognostic significance of sEV-derived ncRNAs in PDAC

Reference	Sample	sEV Isolation	Normalization	ncRNA	Tumor type	Patients by UICC stage					Effect	UVA	MVA
						I	IIA	IIB	III	IV			
Mikamori et al. [127]	Plasma	EQ	cel-miR-39	miR-155	PDAC	23			–	–	High expression correlated with shorter DFS	✓	–
Takahasi et al. [175]	Plasma	UC	miR-16a	miR-451a	PDAC	7	43		–	–	High expression correlated with shorter DFS/OS	✓	✓
Goto et al. [145]	Serum	EQ	*not specified*	miR-21	all PC	2	7	4	5	14	High expression correlated with shorter OS (344 days vs. 846 days)	✓	✓
Li et al. [53]	Plasma	EQ	RNU6, β-actin mRNA	lncSox2ot	PDAC	28		28			High expression correlated with shorter OS (12 months vs. 15 months)	✓	✓
Li et al. [47]	Plasma	EQ	RNU6, β-actin mRNA	circPDE8A	PDAC	47		40			High expression correlated with shorter OS (13 months vs. 16 months)	✓	✓
Wang et al. [84]	Serum	TEI	cel-miR-39	miR-301a-3p	all PC	20			30		High expression correlated with shorter OS	✓	✓
Li et al. [57]	Plasma	TEI	*not specified*	circIARS	PDAC	42		39			High expression correlated with shorter OS (11 months vs. 16 months)	✓	✓
Li et al. [49]	Plasma	EQ	*not specified*	miR-222	PDAC	39		34			High expression correlated with shorter OS (10 months vs. 17 months)	✓	✓
Kawamura et al. [161]	PVB	UC	cel-miR-39, miR-16	miRs 21, 451a, 4525	PDAC	4	27	24	–	–	High expression correlated with shorter DFS/OS	✓	✓
Reese et al. [155]	Serum	UC	cel-miR-39	miR-200c	PDAC	–	4	14	22	16	High expression correlated with shorter OS (miR-200c: 11 months vs. 18 months) (miR-200b: 9 months vs. 18 months)	✓	–
				miR-200b								✓	✓
Guo et al. [116]	Serum	EQ	β-actin	lncUCA1	all PC	20			26		High expression correlated with shorter OS	✓	–
Shao et al	Serum	EQ	cel-miR-39	miR-483-3p	PDAC	18	37		6	2	High expression correlated with shorter OS	✓	✓
Nishiwada et al. [176]	Plasma/Serum	TEI	miR-16	6 miR-panel (130b-5p, 133a-3p, 195-5p, 432-5p, 1229-3p, 1273f)	PDAC	3	8	14	–	–	*Discovery cohort* “high risk” correlated with shorter RfS	✓	✓
						9	12	33	26	2	*Training cohort* “high risk” correlated with shorter RfS	✓	✓
						3	22	31	1	–	*Validation cohort I (did not receive NAT)* “high risk” correlated with shorter RfS	✓	✓
						10	21	13	2	–	*Validation cohort II (received NAT)* “high risk” correlated with shorter RfS	✓	✓

cel, Caenorhabditis elegans; DFS, disease-free survival; EQ, ExoQuick (*SystemBiosciences*); MVA, multivariate survival analysis; n, number of patients; NAT, neoadjuvant therapy; OS, overall survival; PC, pancreatic cancer; PDAC, pancreatic ductal adenocarcinoma; PVB, portal vein blood; TEI, Total Exosome Isolation Kit (*ThermoFisher*); UC, ultracentrifugation; UVA, univariate survival analysis

### Diagnostic significance of sEV-ncRNAs

For the first time in 2013, Que et al. reported about the deregulation of certain sEV-ncRNAs in the serum of PDAC patients, finding that serum-derived sEV-miRNAs 17-5p and 21 were significantly upregulated in patients with PDAC and could distinguish these from healthy controls (HC) and patients with other pancreatic diseases with an area under the receiver operating characteristic (ROC) curve (AUC) of 0.887 (miRNA-17-5p) and 0.897 (miRNA-21) [142]. Ever since, miRNA-21—a known oncogene—has been the sEV-miRNA most frequently evaluated as a diagnostic marker in PDAC [143]. In other studies, its diagnostic accuracy as an individual serum sEV biomarker was calculated as 82.6% or 83%, which could be optimized to 90% when combining miRNA-21 with miRNA-210 or carbohydrate antigen 19-9 (CA.19-9) [144, 145]. In Lai et al., plasma-derived sEV miRNA-21 as well as sEV-miRNAs 10b, 30c, 181a, and let7a could differentiate PDAC from HCs with 100% sensitivity and 100% specificity [146]. It should be noted, however, that the cohort of PDAC patients consisted almost exclusively of patients with UICC stage IIB PDAC. Moreover, Lai et al. used miRNA-425-5p for normalization of microRNA expression, which has since been described to be deregulated in tissue, serum, and serum sEVs of cancer patients, including patients with PDAC [147–149].

While the majority of researchers have used conventional methods for isolation of sEVs and relative quantification of ncRNAs, microfluidics-based approaches have recently become increasingly popular. Using a microfluidic chip that allowed for simultaneous isolation of sEVs and quantification of sEV-derived biomarkers, miRNA-21 could differentiate between PDAC and HCs with a diagnostic accuracy of 93.9% using a sample volume of only 2 µl [150]. The authors also assessed the diagnostic accuracy of miRNAs 10b and 451a, which was 87.5% and 93.0%, respectively. More importantly, however, when considering a biomarker panel consisting of all three miRNAs in combination with EphA2, that biomarker panel could diagnose early-stage PC (UICC I + II), late-stage PC (UICC III + IV), and HCs with 100% accuracy [150]. In a similar approach, Pu et al. applied a tethered cationic lipoplex nanoparticle biochip for absolute quantification of microRNAs using fluorescing molecular beacons [151]. In their study, miRNA-21 and miRNA-10b could differentiate between PC patients and HCs with an accuracy of 71.7% and 65.4%, respectively, while combining both miRNAs entailed an approved accuracy of 79.1% [151].

Apart from miRNA-21, several other sEV-miRNAs have been attributed diagnostic potential in PDAC, although only few have been evaluated in more than one study, in part with contradictory results. For example, in the aforementioned study by Lai and colleagues, sEV miRNA-122 was described as being downregulated in PDAC with an AUC of 0.99, while Zhou et al. found miRNA-122 to be overexpressed in sEVs of PC patients with an AUC of 0.772 [146, 152]. Both research groups used different methods for isolation of sEVs and normalization of miRNA expression. Moreover, Lai et al. solely enrolled patients with PDAC, while Zhou et al. proposedly enrolled all subtypes of PC. Anyhow, these diverging results underline the requirement of larger, multicenter studies for the establishment and validation of novel biomarkers.

The diagnostic potential of singular ncRNA-based biomarkers is often limited and might also be heavily influenced by for example inflammatory conditions [153]. Hence, combining sEV-ncRNAs has proven to be a valuable strategy for a more accurate diagnosis of PDAC. Zhou et al. found the diagnostic accuracy of plasma sEV-miRNAs 122-5p and 193b-3p to be only 77.2% and 65.1%, respectively, while the combination of both yielded an accuracy of 84.9% [152]. Furthermore, Shao et al. suggested combining sEV-derived and circulating miRNAs for a more reliable diagnosis of PDAC. Diagnosis of PDAC with serum sEV miRNA-483-3p was only 69% accurate, while combining it with circulating miRNA-483-3p improved the accuracy to 83% [154].

Recently, a biomarker panel consisting of CA.19-9 as well as miRNAs 200b and 200c derived from total serum sEVs and EpCAM (epithelial cell adhesion molecule)-positive serum sEVs was evaluated. Its diagnostic accuracy in differentiating between PDAC and non-PDAC was 97%, although the diagnostic accuracy for any of the miRNAs fractions alone did not exceed 77% [155]. Moreover, a combination of four miRNAs (1246, 4644, 3976, and 4306) was found to be able to distinguish PDAC from non-PDAC with a sensitivity of 81% and specificity of 93% [156]. Interestingly, the diagnostic potential of aforementioned miRNA-1246 as well as that of miRNAs 196a and 196b was also assessed in early-stage PC only (UICC stages I & IIA), achieving decent AUC values of 0.73 (miRNA-1246), 0.81 (miRNA-196a), and 0.71 (miRNA-196b) [157]. Unfortunately, however, the three miRNAs were not evaluated as a biomarker panel, calling for larger follow-up studies, as diagnosis of PC at early stages is especially difficult to achieve but could offer significant prognostic benefits.

While the majority of studies have been dedicated towards the evaluation of serum or plasma-derived sEV-ncRNAs, sEV are being actively secreted in almost every bodily fluid. Hence, a small number of studies have analyzed sEV-miRNAs expression in clinical specimens other than blood. Expression of miRNA-1246 and miRNA-4644 derived from saliva sEVs could distinguish between patients with pancreaticobiliary malignancies and HCs with an AUC of 0.814 and 0.763, respectively, while the combination of both miRNAs yielded a slightly improved AUC of 0.833 [158]. In contrast, Nakamura et al. quantified the expression of sEV-miRNAs 21 and 155 in pancreatic juice, which could differentiate between PDAC and CP patients with 83% (miRNA-21), 89% (miRNA-155), and 91% accuracy (miRNAs 21 + 155), while Yoshizawa et al. found that the ratio of miRNAs 3940-5p and 8069 in urine sEVs was predictive of PDAC and could distinguish PDAC from CP patients and HCs with an AUC of 0.732 [159, 160]. Interestingly, Kawamura et al. reported that using portal vein blood sEVs for quantification of miRNAs allowed for a more accurate diagnosis of PDAC than using peripheral blood sEVs (AUCs: miRNA-21: 0.727 vs. 0.582; miRNA-451a: 0.745 vs. 0.655; miRNA-4525: 0.836 vs. 0.618) [161]. While the clinical advantage of this proposed method for diagnosis of PDAC remains questionable due to its increased invasiveness, the authors also evaluated the prognostic significance of said miRNAs, which we have discussed separately.

With studies on miRNAs accounting for the vast majority of trials, lncRNAs HULC and UCA1 currently are the only non-miRNA ncRNAs that have been assessed in regard to their diagnostic potential in PC. lncRNA-HULC could distinguish PDAC patients from non-PDAC patients with 92% accuracy, while lncUCA1 presented with a diagnostic accuracy of 78.1% [56, 116]. On a final note, Kitagawa et al. assessed the potential of several sEV-snoRNAs concerning their ability to differentiate PDAC patients from HCs [162]. The determined AUC values were 0.909 (SNORA74A), 0.903 (SNORA25), 0.883 (SNORA22), 0.875 (SNORA14B), and 0.862 (SNORD22). The authors did not specify, however, which method of sEV isolation or which gene for normalization of ncRNA expression was used, leaving questions in regard to the methodological validity of the study [162].

Continuous improvements are being made in standardizing the isolation and characterization of sEVs which previously have always posed major hurdles in establishing sEVs for clinical use. Accordingly, a first exosome-based test has been implemented by the National Comprehensive Cancer Network (NCCN) prostate cancer screening guidelines and has been granted FDA breakthrough designation [163–166]. In PDAC, however, the majority of diagnostic biomarker studies investigating sEV-ncRNAs have been retrospective monocenter trials examining single biomarkers with in part contradictory results [146, 152]. Although most of these single ncRNAs presented with excellent diagnostic accuracy, actual clinical significance seems unlikely, given the multifactorial regulation of ncRNAs. Therefore, future trials investigating sEVs as liquid biopsies should not be limited to the evaluation of single nucleic acid-based biomarkers. Much rather, the combination of sEV-derived nucleic acids and proteins has been shown to significantly enhance accurate diagnosis of cancer, including PDAC [167, 168]. Non-invasive diagnosis of PDAC—especially at early, localized stages—is difficult to achieve. However, in light of the considerable improvements being made regarding the standardization of sEV isolation as well as cost-efficiency of proteomic analysis and nucleic acid sequencing, the addition of liquid biopsies to the diagnostic algorithm of PDAC seems more tangible than ever.

### Prognostic significance of sEV-ncRNAs

Over the last decades, the prognosis of patients with PDAC has not improved significantly and the current 5-year relative survival rate is still below 10% [1]. Bearing in mind that less than 20% of PDAC patients undergo surgery in curative intent, the dismal prognosis can largely be attributed to the failure of multiple late-stage clinical trials investigating novel targeted therapies and immunotherapy in PDAC that in contrast have revolutionized the treatment of many other tumor types [67, 68, 121, 169–172]. As a consequence, systemic therapy of PDAC still heavily relies on chemotherapeutic regimens. Rarely offering substantial improvements in terms of overall prognosis, these therapies can entail considerable side effects [173, 174]. Thus, risk stratification of patients—especially of those with unresectable disease—is becoming increasingly important and should routinely be considered when deciding on the intensity of treatment, follow-up, and aftercare. In that matter, several studies have recently highlighted sEV-ncRNAs as potential prognostic biomarkers in PDAC (Table 3). In 2015, researchers found that low expression of plasma sEV miRNA-155 was associated with prolonged disease-free survival in curatively resected patients [127]. Similarly, low expression of serum sEV-miRNAs 301a-3p, 222, 200c, 483-3p was later reported to be associated with prolonged overall survival of PDAC patients, too [49, 84, 154, 155]. In contrast, miRNA-200b from total serum sEVs was not predictive of overall survival of PDAC patients, however, looking at the subfraction of serum sEVs with surface expression of EpCAM, low expression of miRNA-200b was associated with prolonged overall survival in both all PDAC patients and the subgroup of PDAC patients undergoing surgery in curative intent [155].

In PDAC patients with resectable disease, expression of plasma sEV miRNA-451a was inversely correlated with disease-free survival and overall survival [175]. Furthermore, low expression of miRNA-21 derived from serum sEVs was also found to be associated with a significant benefit in overall survival [145]. Interestingly, Kawamura and colleagues later investigated both sEV-miRNAs 451a and 21 as well as 4525—this time derived from intraoperative portal vein blood of patients undergoing surgery for PDAC—and high expression of all three miRNAs was associated with a significant disadvantage in regard to both disease-free and overall survival [161].

Concerning sEV-lncRNAs and sEV-circRNAs, Li et al. found that the expression of plasma sEV lncRNA-Sox2ot could potentially serve as an independent prognostic marker for overall survival [53]. Similar results were obtained for circRNAs PDE8A and IARS. Researchers found that both circRNAs could potentially serve as independent prognostic markers for PDAC, as overexpression of circRNA-PDE8A and circRNA-IARS was associated with a significant disadvantage in overall survival of patients [47, 57]. On a final note, high expression of sEV lncRNA-UCA1 was associated with shorter overall survival, too [116].

Altogether, the above-reviewed studies underline a general prognostic potential of sEV-ncRNAs, although actual clinical utility of the ncRNAs examined above is likely limited due to the same reasons mentioned before. Evaluating combinations of biomarkers is becoming increasingly popular in a diagnostic setting. Nonetheless, all of the studies summarized above have investigated singular biomarkers. As an exception, researchers recently identified several sEV-miRNAs by transcriptome profiling. As a panel, these miRNAs were capable of predicting PDAC recurrence following surgery in curative intent [176]. Importantly, the biomarker panel exhibited reasonable prognostic accuracy in four separate patient cohorts, providing a sound basis for verification in larger, prospective clinical trials.

## sEVs as delivery vehicles for ncRNA-based therapy in PDAC

**Fig. 4:**
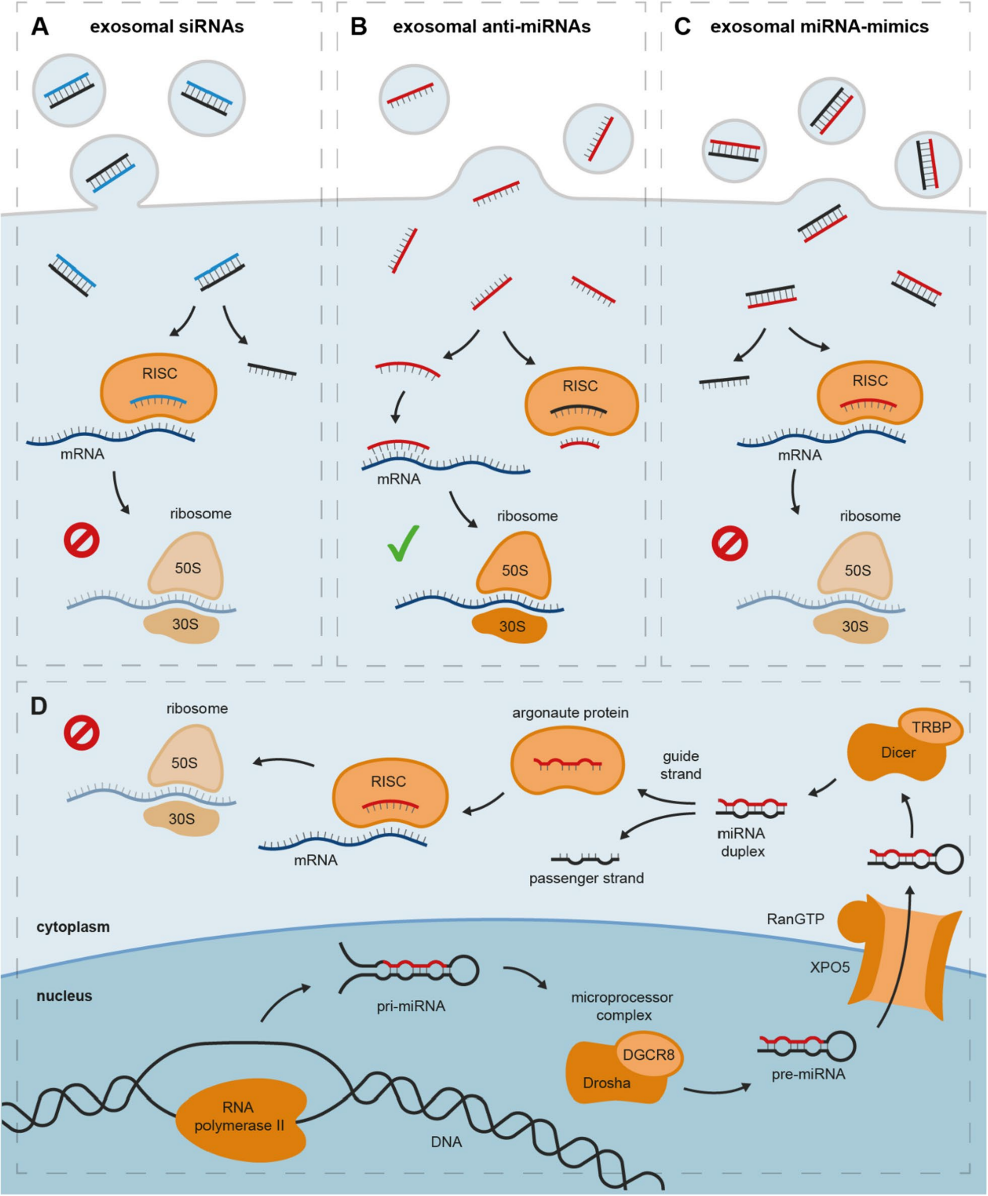
Exosomal ncRNAs as cancer therapeutics and physiological miRNA-mediated gene silencing. **A** Exosomal siRNAs inhibit the translation of oncogenic proteins by mRNA cleavage; **B** exosomal anti-miRNAs enable the translation of tumor-suppressive proteins by inhibiting the interaction of mRNA and RISC through (1) binding the untranslated region of mRNA or 2) directly binding the RISC; **C** exosomal miRNA-mimics inhibit the translation of oncogenic proteins through mRNA cleavage or translational repression. **D** Physiological miRNA-mediated gene silencing. *DGCR8* DiGeorge syndrome critical region 8, *pre-miRNA* precursor microRNA, *pri-miRNA* primary microRNA, *RISC* RNA-induced silencing complex, *TRBP* transactivation response element RNA-binding protein, *XPO5* exportin 5

Over the last two decades, considerable advances have been made in utilizing ncRNAs for the treatment of cancer. The majority of ncRNA-based therapies capitalize on a mechanism known as RNA interference (RNAi). In humans, RNAi is physiologically mediated through miRNAs (Fig. 4D). In short, pri-miRNAs are initially transcribed from miRNA genes by RNA polymerase II and further processed in the nucleus to pre-miRNAs by an enzyme complex known as the microprocessor complex that consists of RNA-binding protein DGCR8 and RNase III-type protein Drosha [177, 178]. pre-miRNAs are then actively transported into the cytoplasm by Exportin-5 in a RanGTP-dependent manner [179]. In the cytoplasm, they are further cropped by RNase III-type protein Dicer with the help of TRBP to yield a miRNA duplex [180]. The miRNA duplex—consisting of a guide strand and a passenger strand—is then loaded onto argonaute proteins [181, 182]. Ejecting the passenger strand, the miRNA guide strand and argonaute proteins form the mature RISC that can bind complementary mRNA and induce translational repression or mRNA decay [183]. In cancer, RISC-mediated gene silencing is often thrown off balance: overexpression of oncogenic miRNAs can lead to excessive repression of tumor-suppressive proteins, while inhibition of tumor-suppressive miRNAs can entail overexpression of oncogenic proteins.

Today, RNAi-based cancer therapeutics can be categorized into three groups: small interfering RNAs (siRNAs), miRNA-mimics, and anti-miRNAs. (1) siRNAs are double-stranded RNA molecules with perfect complementarity to a specific mRNA. Similar to miRNAs, siRNAs are loaded onto argonaute proteins in the cytoplasm. Following removal of the passenger strand, siRNAs and argonaute proteins form the RISC, allowing them to exert influence on gene expression through mRNA cleavage (Fig. 4A). (2) anti-miRNAs aim at silencing endogenous oncogenic miRNAs. anti-miRNAs can either bind oncogenic miRNAs or the untranslated region of complementary target mRNAs, thus preventing the interaction of RISCs and mRNAs. This enables mRNAs to be translated into tumor-suppressive proteins (Fig. 4B) [184–186]. (3) In contrast, it has frequently been shown that loss of tumor-suppressive miRNAs can contribute to carcinogenesis and tumor progression. Hence, miRNA-mimics act as a miRNA replacement therapy that—once loaded onto argonaute proteins—can effectively suppress the translation of oncogenes (Fig. 4C) [187–189].

Translation of RNAi-based therapies into the clinic has faced several challenges. Systemically delivered miRNAs and siRNAs face degradation by nuclease activity and are rapidly cleared from circulation via the kidneys. Moreover, low levels of endosomal escape can greatly reduce the transfection efficiency of tumor cells after endosomal incorporation of the therapeutic payload [190].

Facing these challenges, sEVs have recently emerged as promising vehicles for efficient tumor-directed delivery of miRNA- and siRNA-based therapeutics. As an endogenous form of intercellular communication, sEVs have been shown to be well-tolerable with low immunogenicity [191, 192]. Moreover, researchers have shown that sEVs have the ability to escape phagocytosis by monocytes and macrophages as well as complement-mediated lysis by surface expression of CD47 and CD55/59, respectively, greatly increasing their time of circulation in the bloodstream [193, 194]. To enhance intratumoral accumulation while simultaneously reducing therapy-related side effects, researchers have shown that sEVs can be engineered to specifically target tumor tissue [195–199]. And in PDAC, specifically, Kyuno et al. have recently shown that tailoring sEVs to express tetraspanin 8 greatly improved their uptake in PDAC cells in vivo [200].

The prospect of sEV-encapsulated ncRNA-therapeutics has led to the initiation of several preclinical studies in PDAC (Table 4). In 2019, Ding et al. overexpressed miRNA-145-5p in sEVs derived from human umbilical cord mesenchymal stem cells by transfection [201]. Intratumoral injection of said sEVs resulted in a significant reduction of tumor growth in vivo. Authors showed that the tumor-suppressive function of miRNA-145-5p could be attributed to direct downregulation of Smad3, a mediator of TGF-β signaling, frequently altered in PDAC [81, 201]. Wu and colleagues used bone marrow mesenchymal stem cells (BM-MSCs) for the production of therapeutic sEVs [202]. Previous studies had shown that miRNA-126 could inhibit PDAC progression through downregulation of ADAM9 [203]. Therefore, Wu and colleagues induced overexpression of miRNA-126-3p in BM-MSCs which led to an enrichment of miRNA-126-3p in BM-MSC sEVs. sEV-mediated delivery of miRNA-126-3p to PDAC xenograft tumors in vivo resulted in downregulation of ADAM9 and subsequent inhibition of tumor progression [202]. With their potential for immortalization, mesenchymal stem cells are a popular choice for the continuous large-scale production of sEVs. Similarly, Shang and colleagues also used BM-MSCs as sEV donors [204]. sEVs enriched with miRNA-1231 through transfection of BM-MSCs were injected into BALB/c nude mice harboring BxPC-3 xenografts, which led to a significant reduction of tumor weight and volume [204]. Moreover, incubation of PDAC cells with sEV-encapsulated miR-124-3p-mimic prior to their injection into mice was shown to enhance the anti-tumor activity of 5-FU (fluorouracil) in a subcutaneous xenograft later on [205].

**Table 4:** Preclinical trials of sEV-encapsulated ncRNAs as therapeutics in PDAC

Ref	sEV donor	Cargo loading	ncRNA	Mouse model	Therapeutic intervention	Result
Kamerkar et al. [193]	Human foreskin fibroblast	Electroporation	siKRAS^G12D^	orthotopic PANC-1/BxPC-3/KPC689 xenografts	Intraperitoneal injection of sEVs	Prolonged survival, inhibition of metastasis
				KPC/KTC GEMM		
Mendt et al. [206]	BM-MSC	Electroporation	siKRAS^G12D^	Orthotopic KPC689/PANC-1 xenograft	Intraperitoneal injection of sEVs	Prolonged survival, enhanced effect of gemcitabine
				Orthotopic PATX-60 PDX		
				PKS GEMM		
Ding et al. [201]	HUC-MSC	Transfection	miR-145-5p mimic	Subcutaneous PANC-1 xenograft	Intratumoral injection of sEVs	Reduced tumor growth
Wu et al. [202]	BM-MSC	Transfection	miR-126-3p mimic	Subcutaneous xenograft	Co-injection of BM-MSCs	Reduced tumor growth
Shang et al. [204]	BM-MSC	Transfection	miR-1231 mimic	Subcutaneous BxPC-3 xenograft	Tail vein injection of sEVs	Reduced tumor growth
Zuo et al. [207]	HEK293	Ultrasound	miR-34a mimic	Subcutaneous Panc28 xenograft	Intravenous injection of sEVs	Reduced tumor growth
Xu et al. [208]	PANC-1	Electroporation	siPAK4	Subcutaneous PANC-1 xenograft	Intratumoral injection of sEVs	Reduced tumor growth
Zhou et al. [209]	BM-MSC	Electroporation	siGalectin9	Orthotopic PANC-02 xenograft	Tail vein injection of sEVs	Enhanced effect of immunotherapy

5-FU, fluorouracil; BM, bone marrow; GEMM, genetically engineered mouse model; HUC, human umbilical cord; MSC, mesenchymal stem cell; PDX, patient-derived xenograft; Ref., reference

In contrast, Shi et al. used normal fibroblast sEVs for delivery of miRNA-520b to PDAC cells, successfully decelerating tumor growth and metastatic dissemination in vitro, while co-incubation of PDAC cells with miRNA-520b-overexpressing normal fibroblast sEVs prior to subcutaneous injection in mice resulted in decelerated tumor growth and reduction of the number of metastases [210]. In addition to the aforementioned miRNAs, several sEV siRNAs have been evaluated in light of their therapeutic potential in PDAC.

PAK4—a member of the PAK family of serine threonine kinases, linked with PI3K/AKT and Wnt/β-catenin signaling—has been identified as a promising therapeutic target in cancer [211, 212]. With two small molecule inhibitors of PAK4 currently being evaluated in Phase I basket trials in solid malignancies and non-Hodgkin lymphoma (NCT04281420, NCT02702492), Xu et al. recently reported that PAK4 could also be targeted by sEV-siRNA [208]. Authors loaded PDAC-derived sEVs with PAK4-targeted siRNA (siPAK4) by electroporation. Subsequent intratumoral injection of siPAK4 sEVs into murine PANC-1 xenografts proved to be highly efficient in slowing down tumor growth and prolonging overall survival of mice, while at the same time exhibiting a tolerable safety profile in regard to liver toxicity [208]. It should be noted, however, that although tumor sEVs have been shown to distribute preferentially to tumor tissue, the utilization of tumor sEVs as delivery vehicles seems questionable in a clinical setting [213]. As opposed to for example melanoma and lung cancer, PDAC is thought to be an immunologically ‘cold’ tumor, resulting in limited success of immunotherapy [214]. Recently, Galectin-9 was identified as a novel component of the multifaceted network contributing to the immunosuppressive TME of PDAC [215, 216]. Zhou et al. co-loaded BM-MSC-derived sEVs with Galectin-9-specific siRNA and oxaliplatin [209]. Administration of these sEVs significantly improved overall survival in an orthotopic murine PDAC xenograft. Furthermore, e*x vivo* analysis of murine PDAC tumors revealed a reduction in the ratio of M2 to M1-polarized TAMs, a potential correlate of the successful reprogramming of the immunosuppressive PDAC TME [209].

In contrast to Galectin-9, the central role of KRAS in carcinogenesis and progression of cancer has been known for decades, even though KRAS had long been dubbed an ‘undruggable’ target. KRAS—a key downstream GTPase of several growth factor receptors and member of the RAS family of GTPases—is frequently mutated in multiple tumor entities including lung adenocarcinoma (33%), colorectal carcinoma (42%), and PDAC (93%) [217–219]. Recently, promising results from preclinical and early-stage clinical trials of two small molecule inhibitors, MRTX-849 (‘adagrasib’) and AMG-510 (‘sotorasib’), have renewed hopes that KRAS can be targeted after all [220–223]. In a similar approach, Kalluri and colleagues engineered siRNA specifically targeting G12D-mutated KRAS (KRAS^G12D^, siKRAS^G12D^), the predominant alteration of KRAS in PC [193]. Authors encapsulated siKRAS^G12D^ in sEVs (‘iExosomes’) and liposomes (‘iLiposomes’) and then evaluated their potential as a therapy for PDAC. iExosomes in particular showed promising anti-tumor activity. Systemic iExosome treatment in xenografts and genetically engineered mouse models of PDAC led to a remarkable suppression of tumor growth, prolonged survival, and inhibition of metastasis. In that regard, iExosomes were far superior to iLiposomes, which was mainly attributed to two aspects: (1) CD47 expression on exosomes inhibited phagocytosis by monocytes, thus prolonging their time of circulation and (2) oncogenic KRAS induced macropinocytosis-mediated uptake of exosomes in tumor tissue [193]. These results have since triggered the initiation of a Phase I clinical trial of iExosomes in 28 patients with metastatic PDAC harboring G12D mutations of KRAS (NCT03608631).

In 2016, a Phase I clinical trial (NCT01829971) investigating MRX34, a miRNA-34a-mimic administered in a liposomal formulation, was prematurely terminated due to immune-mediated toxicity that resulted in the death of four patients [187]. Researchers have since evaluated how the safety of miR-34a-mimics as cancer therapeutics could be improved, generally commending their potential as tumor-suppressors [224]. In that matter, immunogenicity of delivery vehicles and efficient delivery to the tumor tissue have been identified as key challenges that need to be addressed [224]. Notably, Zuo et al. recently utilized sEVs instead of liposomes for miR-34a delivery to PC cells, yielding promising anti-tumor activity in a preclinical setting, potentially paving the way for further studies on miR-34a as a novel therapeutic in PDAC [207].

While utilizing sEVs for the delivery of therapeutic ncRNAs solves many problems such as immunogenicity, tumor-directed delivery as well as stability in the bloodstream that are encountered with many other delivery vehicles, the major bottlenecks for exosome therapeutics are (1) reliable, large-scale production of exosomes and (2) efficient loading of the therapeutic payload into exosomes. Kalluri and colleagues have addressed this problem by providing a protocol for large-scale production of exosomes in line with good manufacturing practice standards and efficient siRNA-loading of exosomes by electroporation [206]. Moreover, pharmaceutical companies have started to investigate the potential of exosome-based therapeutics in (pre-)clinical programs and several companies have recently reported encouraging preclinical data in cancer [225, 226]. Given their endogenous nature as intercellular carriers of genetic information, the choice of exosomes as therapy vehicles for ncRNAs seems obvious. It took several years for the first ncRNA-based therapy to be approved. Therefore, combining and further pursuing research on exosomes and ncRNAs in the future will hopefully allow researchers to fully capitalize on both technologies in PDAC and beyond.

## Conclusions and future directions

In recent years, extensive research on sEV-ncRNAs has greatly progressed our understanding of PDAC. Several studies have highlighted the significance of sEV-ncRNAs as liquid biopsies. Even so, major challenges remain to be solved prior to the implementation of sEV-ncRNAs as clinical assays in PDAC. As is common practice in biomarker discovery, the vast majority of the initial studies on sEV-ncRNAs have been non-randomized retrospective single-center studies enrolling less than 100 patients. Although these studies serve as proof of concept, the clinical applicability of the majority of identified biomarkers is likely limited. In future, sEV transcriptome sequencing and data submission to public repositories should be applied more readily to accelerate the identification of further biomarker candidates. Moreover, during transition to biomarker validation, conduction of larger, randomized, prospectively enrolling multicenter trials will be necessary to identify those sEV-ncRNAs, from which a general patient population could profit.

Deregulation of sEV-ncRNAs has not only been observed in cancer but also in multiple other diseases. Furthermore, differential expression of sEV-ncRNAs might also be subject to environmental factors and patient characteristics such as age, gender, and ethnicity. Therefore, the utility of single ncRNA-based biomarkers is presumably limited. In future, evaluation of biomarker panels is advisable and will likely yield more viable results, while composition of these panels need not be limited to sEV-ncRNAs but should include mRNA, proteins, and DNA as well [167]. Given their tissue-specific heterogeneity, identification of disease-specific surface markers should also be evaluated to fully exploit the potential of sEVs in PDAC [227].

While identification of suitable biomarkers itself is crucial, standardization and scalability are obligatory requirements for clinical application and these are especially challenging in EV research. sEV-ncRNA analysis is time-consuming and requires multiple work steps, most noticeably sEV separation and RNA quantification. While RNA quantification by quantitative polymerase chain reaction is an inherent part of laboratory diagnostics, at least nine different methods are recurrently used by researchers for sEV separation [33]. More importantly, due to varying functionality, these methods have been shown to enrich varying subpopulations of sEVs with potentially different content [228]. Therefore, scalability of methods for use in clinical routine should ideally be brought into question prior to devoting resources on large validation studies.

On a molecular level, multiple studies have investigated the role of sEVs as carriers of ncRNAs for intercellular signaling, which has provided us with a deeper understanding of PDAC. Given the large body of literature, an involvement of sEV-ncRNAs in cancer hallmarks such as immunosuppression, angiogenesis, therapeutic resistance, and metastatic dissemination seems likely. It should be noted, however, that most of the studies administered sEVs in supraphysiological concentrations without examining the dose-dependency of the observed effects, which should be addressed in the future more frequently. Moreover, adjusting environmental factors such as pH and oxygen level to conditions commonly seen in the TME could further enhance clinical applicability of observations. Regardless of dose-dependency and environmental factors, these studies have confirmed the oncogenic as well as tumor-suppressive nature of deregulated ncRNAs in PDAC and this has led to the initiation of several preclinical trials investigating sEV-ncRNAs as cancer therapeutics. Moreover, one clinical-stage trial is currently investigating exosome-encapsulated KRAS-directed siRNA in PDAC and its read-out—expected in March 2022—will be a guideline for future research on EV therapeutics.

Altogether, sEV-ncRNA research faces great challenges that are particularly of technical nature. Given the potential of sEV-ncRNAs as liquid biopsies and therapeutics, resolving these issues seems worth the effort, as sEV-ncRNAs could significantly contribute to the multi-modal approach that will be necessary to overcome PDAC.

## Data Availability

The datasets analyzed during the current study are available in the US National Library of Medicine repository (https://clinicaltrials.gov).

## Abbreviations

AUC: Area under the ROC curve
BM-MSC: Bone marrow mesenchymal stem cell
CA.19-9: Carbohydrate antigen 19-9
CAF: Cancer-associated fibroblast
ceRNA: Competitive endogenous RNA
circRNA: Circular RNA
EMT: Epithelial-to-mesenchymal transition
ESCRT: Endosomal sorting complex required for transport
EV: Extracellular vesicle
GEM: Gemcitabine
iExosomes: Tailored sEVs containing siKRAS^G12D^
iLiposomes: Tailored liposomes containing siKRAS^G12D^
ILV: Intraluminal vesicle
lncRNA: Long non-coding RNA
miRNA: Micro RNA
mRNA: Messenger RNA
MSC: Mesenchymal stem cell
MVB: Multivesicular body
ncRNA: Non-coding RNA
PC: Pancreatic cancer
PDAC: Pancreatic ductal adenocarcinoma
PM: Plasma membrane
PSC: Pancreatic stellate cell
RISC: RNA-induced silencing complex
ROC: Receiver operating characteristic
rRNA: Ribosomal RNA
sEV: Small extracellular vesicle
sEV-ncRNA: sEV-derived ncRNA
siRNA: Small interfering RNA
siKRASG12D: siRNA targeting G12D-mutated KRAS
snoRNA: Small nucleolar RNA
snRNA: Small nuclear RNA
TAM: Tumor-associated macrophage
TME: Tumor microenvironment
Treg: Regulatory T cell
tRNA: Transfer RNA
